# Targeting of cell-free DNA by *DNase I* diminishes endothelial dysfunction and inflammation in a rat model of cardiopulmonary bypass

**DOI:** 10.1038/s41598-019-55863-8

**Published:** 2019-12-17

**Authors:** Carolyn Weber, Alexander Jenke, Vasilena Chobanova, Mariam Yazdanyar, Agunda Chekhoeva, Kaveh Eghbalzadeh, Artur Lichtenberg, Thorsten Wahlers, Payam Akhyari, Adnana Paunel-Görgülü

**Affiliations:** 1 Department of Cardiothoracic Surgery, Heart Center of the University of Cologne, Cologne, Germany; 2 Department of Cardiovascular Surgery, Heinrich Heine University Düsseldorf, Düsseldorf, Germany

**Keywords:** Inflammation, Translational research

## Abstract

The use of cardiopulmonary bypass (CPB) results in the activation of leukocytes, release of neutrophil extracellular traps (NETs) and severe inflammation. We hypothesize that targeting of circulating cell-free DNA (cfDNA) by *DNases* might represent a feasible therapeutic strategy to limit CPB-associated side effects. Male Wistar rats (n = 24) underwent CPB with deep hypothermic circulatory arrest (DHCA) and were divided into 3 groups: control (group 1), one *i.v*. bolus *DNase I* before CPB start (group 2) and a second *DNase I* dose before reperfusion (group 3). We found a positive correlation between plasma cfDNA/NETs levels and compromised endothelial vasorelaxation after CPB. *DNase I* administration significantly diminished plasma cfDNA/NETs levels. Further, a dose-dependent improvement in endothelial function accompanied by significant reduction of circulating intercellular adhesion molecule (ICAM)-1 was observed. Rats of group 3 had significantly reduced plasma IL-6 levels and downregulated expression of adhesion molecules resulting in impaired leukocyte extravasation and reduced MPO activity in lungs. Mechanistically, digestion of NETs by *DNase I* significantly diminished NETs-dependent upregulation of adhesion molecules in human endothelial cells. Altogether, systemic *DNase I* administration during CPB efficiently reduced cfDNA/NETs-mediated endothelial dysfunction and inflammation and might represents a promising therapeutic strategy for clinical practice.

## Introduction

Cardiac surgery with cardiopulmonary bypass (CPB) initiates a systemic inflammatory response syndrome (SIRS) caused by the surgical trauma itself, blood contact with the non-physiological surfaces of the extracorporeal circuit and ischemia/reperfusion (I/R) injury^1^. During CPB, rapid generation of pro-inflammatory mediators promotes neutrophil activation^2^, and stimulates the release of proteolytic enzymes, antimicrobial proteins and neutrophil extracellular traps (NETs), all of which may mediate deleterious effects to organ systems favoring the development of postoperative complications^3–5^. Recently, a positive correlation between plasma cell-free DNA (cfDNA) and soluble intercellular adhesion molecule-1 (ICAM-1) was found in patients undergoing cardiac surgery with CPB^6^. NETs consist of a chromatin backbone with attached antimicrobial proteins such as histones, myeloperoxidase (MPO), neutrophil elastase and defensin peptides^7^. Many physiological inducers of NETosis have been reported, including activated platelets^8^, activated endothelial cells^9^ and proinflammatory cytokines^10^. Elevated levels of circulating cell-free (cf)DNA/NETs have been observed during I/R injury, inflammation, coagulation, sepsis and notably, in patients undergoing elective cardiac surgery and correlation with perioperative renal dysfunction has been reported^3,11,12^. Endothelial dyfunction in patients undergoing CPB is an important marker of poor outcome^6,13^. Interleukin (IL)-6 and tumor necrosis factor (TNF)-α as well as NETs induce an upregulation of endothelial adhesion molecules, such as ICAM-1, vascular cell adhesion molecule-1 (VCAM-1) or E-selectin^14,15^. Their emergence on the endothelial surface sets the stage for leukocyte-endothelial interactions, which are closely followed by transmigration of activated neutrophils and monocytes into the interstitial space of the reperfused tissue^14^. It has been reported, that critical components of NETs, including neutrophil elastase and MPO, could be responsible for NETs-induced endothelial dyfunction^3,11,16^. Additionally, cytotoxic, pro-inflammatory effects of histones have already been evidenced^17^. As NETs are largely composed of decondensed chromatin, the use of *DNase I* has been suggested to represent a suitable therapeutic option to limit NETs-mediated side effects^18^. Hence, Savchenko *et al*. found that *DNase I* application limits infarct size in a mouse model^19^. Similar findings were reported by Vogel *et al*. who demonstrated *DNase I*-dependent improvement of left ventricular remodeling after myocardial infarction (MI) in mice^20^. Recently, protective effects of *DNase I* in a rat model of intestinal I/R injury have been reported^18^.

However, no studies have so far been conducted to investigate the impact of *DNase I*-based therapies on CPB-induced inflammation and the clinical feasibility of such approaches. Therefore, in the present study, we studied the effects of *DNase I* application on endothelial function and inflammation using a well-established rat model of CPB with deep hypothermic circulatory arrest (DHCA), that was developed to effectively and reproducibly induce global ischemia/reperfusion (I/R), systemic inflammation and injury of organs as major pathogenic factors of a CPB-induced SIRS in a clinic-like setting^21^.

## Results

### Effect of *DNase I* on physiological parameters and compensatory drug application during CPB with DHCA

Blood samples were collected from rats before CPB (T1), before reperfusion (T2) and at the end of reperfusion (T3). The levels of electrolytes, glucose, lactate, O_2_ and CO_2_ are summarized in Table 1. The counts of the major blood cell fractions white blood cells, i.e. red blood cells and platelets did not differ between the control group and *DNase I*-treated animals at T1-T3 (Supplementary Table 1). No differences in haemoglobin concentrations as well as haematocrit values were observed. Finally, the plasma levels of various organ-specific injury markers such as creatinine, urea and uric acid (kidney), aspartate transaminase and alanine transaminase (liver), alpha-amylase (pancreas), neuron-specific enolase (neurons) as well as lactate dehydrogenase at the end of the CPB procedure (T3) were not affected by *DNase I* treatment (Supplementary Table 2). In contrast, cardiac troponin T was not detected in plasma samples of all experimental groups suggesting absence of CPB-induced cardiac injury during and at the end of surgery (not shown).

**Table 1:** *DNase I* does not affect physiological blood parameters during CPB.

	Control			1 × *DNase*			2 × *DNase*		
	T1	T2	T3	T1	T2	T3	T1	T2	T3
Na^+^ (mmol/L)	136.4 ± 0.5	136.1 ± 1.8	140.4 ± 2.8	137.9 ± 3.9	136.7 ± 1.3	140.7 ± 3.9	136.8 ± 1.3	136.9 ± 0.8	139.6 ± 2.7
K^+^ (mmol/L)	3.19 ± 0.63	4.39 ± 0.41*	4.61 ± 1.36*	3.59 ± 0.63	4.23 ± 0.30*	4.70 ± 0.37**	3.46 ± 0.55	4.41 ± 0.29	4.85 ± 0.87**
Ca^2+^ (mmol/L)	0.44 ± 0.17	0.87 ± 0.13***	0.96 ± 0.20***	0.67 ± 0.33	0.93 ± 0.10	0.92 ± 0.16	0.53 ± 0.10	0.95 ± 0.15***	0.94 ± 0.09***
Glucose (mmol/L)	14.6 ± 3.2	22.1 ± 3.2**	19.2 ± 5.7	13.2 ± 3.4	20.8 ± 1.9**	20.7 ± 4.0**	13.4 ± 0.9	20.1 ± 2.7***	20.1 ± 4.3***
Lactate (mmol/L)	1.51 ± 0.47	6.91 ± 2.06***	5.07 ± 2.71**	1.26 ± 0.26	4.97 ± 1.93*	4.93 ± 3.25*	1.28 ± 0.31	5.04 ± 2.04***	3.79 ± 1.52**
pH	7.55 ± 0.02	7.54 ± 0.15	7.17 ± 0.15	7.55 ± 0.03	7.43 ± 0.14	7.20 ± 0.11	7.53 ± 0.09	7.32 ± 0.20	7.11 ± 0.21
sO_2_ (%)	97.1 ± 0.4	97.0 ± 0.4	96.3 ± 3.4	97.0 ± 0.4	96.4 ± 1.2	96.1 ± 0.7	97.0 ± 0.3	96.4 ± 1.1	95.7 ± 0.5
pO_2_ (mmHg)	390.3 ± 110.3	366.3 ± 53.1	325.1 ± 117.8	422.0 ± 69.5	361.7 ± 70.8	320.6 ± 77.6	327.1 ± 94.8	342.0 ± 94.9	278.1 ± 59.1
pCO_2_ (mmHg)	24.5 ± 4.5	22.0 ± 11.2**	60.4 ± 31.7**	25.0 ± 5.0	30.9 ± 14.1	49.4 ± 15.2*	29.0 ± 9.4	46.2 ± 22.1	81.1 ± 39.3*

*T1*, before CPB; *T2*, after CPB and before reperfusion; *T3*, after reperfusion. *p < 0.05; **p < 0.01; ***p < 0.001 versus T1. Data are presented as Mean ± SD. No significant intergroup differences were detected.

Heart rate and MAP values were similar in all experimental groups (Supplementary Fig. 1). For norepinephrine (Arterenol) which was given to prevent a sustained CPB-induced fall of MAP we observed no differences in the amounts applied to animals that received *DNase I* versus controls (Supplementary Fig. 2). Moreover, application of sodium bicarbonate or TRIS (Trometamol) to prevent metabolic acidosis was not necessary throughout the entire CPB procedure in any animal of the examined experimental groups.

### Impact of *DNase I* application on plasma cfDNA levels and plasma *DNase* activity

**Figure 1:**
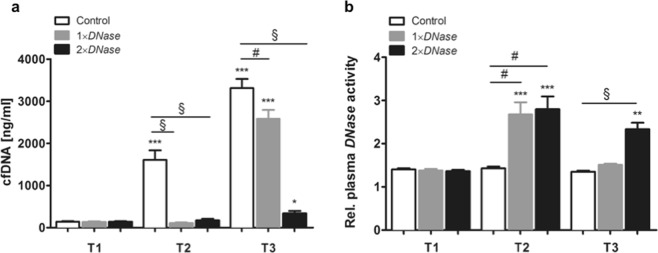
Evaluation of plasma cell-free DNA (cfDNA) levels and *DNase* activity. Rats underwent cardiopulmonary bypass (CPB) with deep hypothermic circulatory arrest (DHCA) as described in the Methods section. Plasma samples were collected from control rats without *DNase I* therapy (n = 7), rats receiving *DNase I* before CPB (1 × *DNase*, n = 7) and those receiving a second *DNase I* dose before reperfusion (2 × *DNase*, n = 8) at following times: before CPB (T1), before reperfusion (T2) and at the end of reperfusion (T3). (**a**) Plasma cfDNA levels were quantified by PicoGreen staining and were found to be significantly decreased upon *DNase I* administration. (**b**) Additionally, relative plasma *DNase* activity was determined and significantly increased in rats that received *DNase I*. **P < 0.01; ***P < 0.001 versus T1; #*P* < 0.01, §*P* < 0.0001.

Plasma levels of cfDNA were quantified at defined times (Fig. 1a). CPB with DHCA strongly increased plasma cfDNA levels reaching a maximum at T3 (24.4-fold increase, *P* < 0.001 vs. T1). It has previously been reported that *DNase I* is rapidly inactivated by serum proteins^22^. Thus, *DNase I* therapy before CPB prevented cfDNA increase at T2 (17-fold reduction vs. Control, *P* < 0.001) but did not influence reperfusion-associated cfDNA elevation at T3. In contrast, two boli of *DNase I*, i.e. before CPB and reperfusion, largely suppressed cfDNA increase at T2 as well as T3 (13-fold reduction vs. Control, *P* < 0.001), indicating that *DNase I* exerts short-term effects. Rats receiving one bolus of *DNase I* displayed increased *DNase* activity only on T2 (1.7-fold increase, *P* < 0.01), whereas the activity at T3 was comparable to that found in control animals without *DNase I* treatment. In contrast, *DNase I* activity was found to be significantly increased after 60 min of reperfusion when the rats received a second bolus of *DNase I* (1.6-fold increase, *P* < 0.001; Fig. 1b).

### Vascular reactivity studies

**Figure 2:**
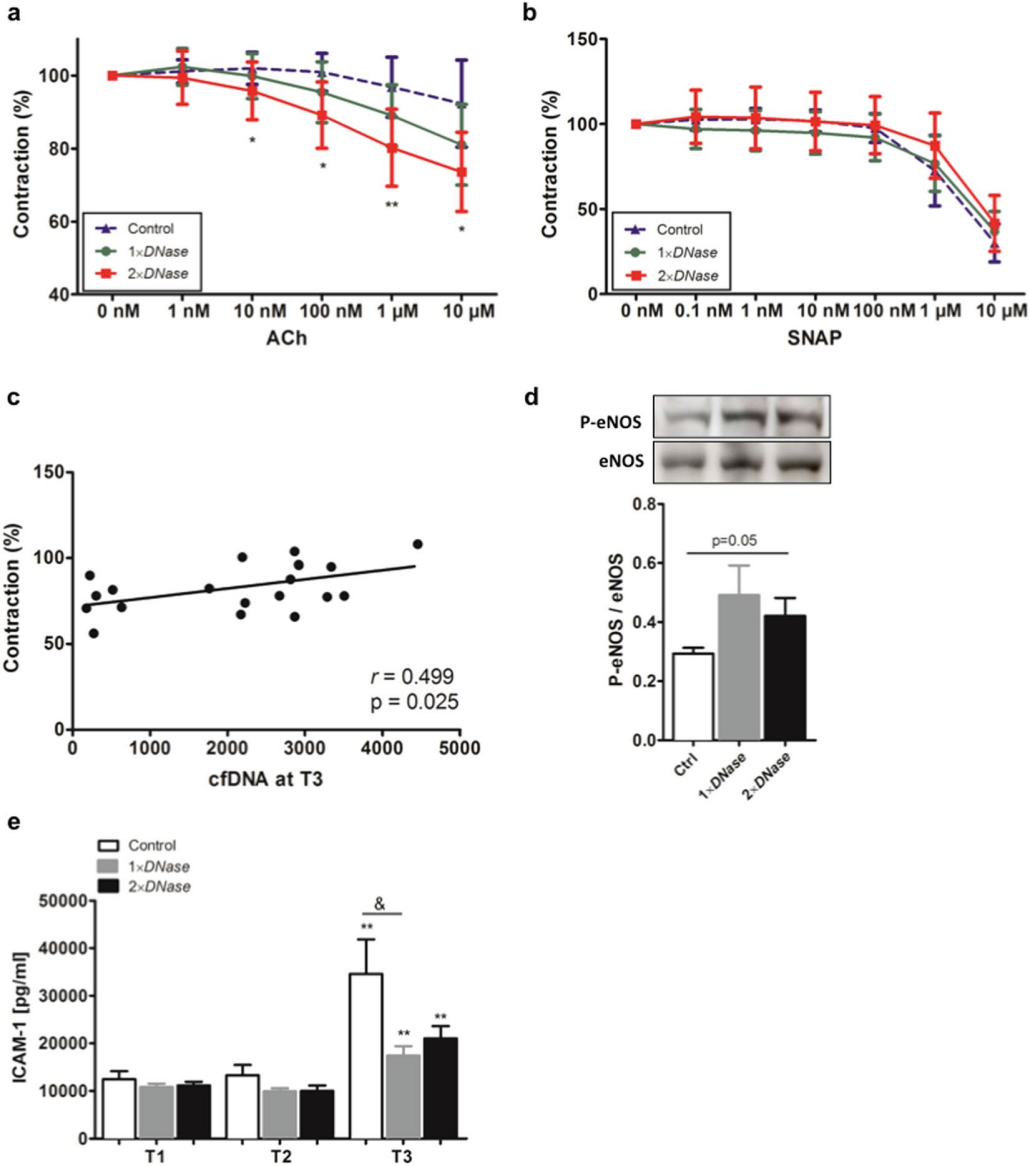
Effects of *DNase I* on vascular function. Rat aortas were removed at the end of surgical procedure and immediately used for *ex vivo* functional analyses. (**a**) Aortic rings (4 mm width) were pre-constricted with 0.1 µM Phenylephrin (PE) and endothelial-dependent vasorelaxation was achieved by the addition of different concentrations of Acethylcholin (ACh, range 0 nM-10 µM). Pre-constriction was defined as 100%. Improved vasorelaxation was found in aortic vessels of rats treated with two doses of *DNase I*. *P < 0.05, **P < 0.001 versus control group. (**b**) To prove endothelium-independent vasorelaxation, aortic rings were first pre-incubated with the NOS inhibitor L-NNA (100 nM) before contraction with 0.1 µM PE and vasorelaxation was induced by the NO donor SNAP (0 nM-10 µM). No differences between groups were found. (**c**) Pearson correlation analysis revealed positive correlation between vasorelaxation induced by 10 µM ACh and cell-free DNA (cfDNA) levels quantified at the end of the reperfusion (T3 = end of surgical procedure). (**d**) Expression of the phosphorylated form of endothelial NO synthase (P-eNOS) in aortic tissue obtained at the end of surgery was quantified by Western blot and normalized to that of total eNOS. Representative cropped western blot images from different blots are depicted. The molecular weight of specific immunolabelled bands is indicated. Samples for the detection of eNOS and P-eNOS were processed and run in parallel. Original images of full-length blots are presented in Supplementary Fig. 3. (**e**) Plasma samples wer**e** collected from rats with or without *DNase I* treatment before cardiopulmonary bypass (CPB) with deep hypothermic cardiac arrest (DHCA) (T1), before reperfusion (T2) and after reperfusion (T3). Levels of soluble intercellular adhesion molecule-1 (ICAM-1) were quantified by ELISA. There was a reduction of ICAM-1 at the end of reperfusion (T3) in plasma collected from rats treated with *DNase I*. **P < 0.01 versus T1; & *P* < 0.05.

To study the effects of cfDNA on the endothelium, functional analyses were performed. Endothelium-dependent vasorelaxation in response to acethylcholine (ACh) was impaired in control rats after CPB with DHCA (Fig. 2a). *DNase I* treatment before CPB slightly improved vasorelaxation whereas significant improvement of vasorelaxation could be observed in aortic rings of rats that received a second dose of *DNase I*. Relaxation to ACh was inhibited in all groups in the presence of the NO synthase inhibitor L-NNA (not shown), confirming the dependence on NO. In turn, endothelium-independent vasorelaxation induced by the NO-donor SNAP was similar in all groups (Fig. 2b).

There was a positive correlation between plasma cfDNA levels at the end of reperfusion (T3) and the extent of vasorelaxation after stimulation with 10 µM ACh (Pearson’s *r* = 0.499, *P* = 0.025; Fig. 2c). In addition, the activity of eNOS, which reflects endothelial function, was lowest in control animals and tended to increase in the aortas of rats receiving *DNase I* (*P* = 0.05; Fig. 2d). The protective effect of *DNase I*-based therapy during CPB on vascular function could be confirmed by determining the levels of circulating ICAM-1 (Fig. 2e). Plasma levels of ICAM-1 were strongly upregulated after reperfusion in all groups and peaked in control animals (2.48-fold increase vs. T1). The CPB-induced upregulation of plasma ICAM-1 levels was significantly attenuated at T3 in animals that received *DNase I* yet no dose dependency was observed (1.9-fold and 1.5-fold reduction in groups 2 and 3, *P* = .04 vs. Control). These results suggest a protective effect of *DNase I* on vascular function.

### Effect of *DNase I* on inflammation

**Figure 3:**
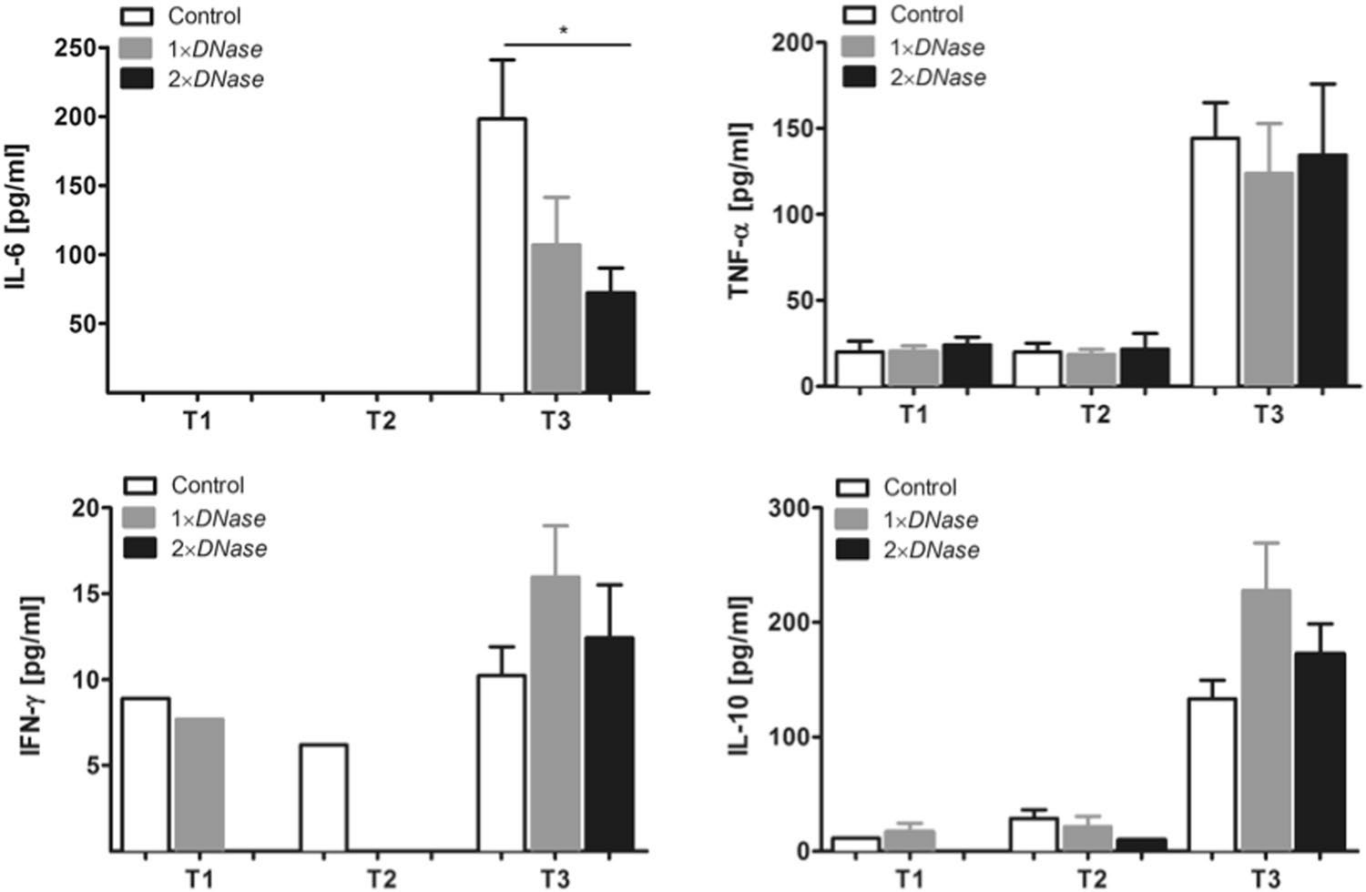
Assessment of *DNase I* effects on plasma cytokine levels. Rats were subjected to cardiopulmonary bypass (CPB) with deep hypothermic cardiac arrest (DHCA) as described in the Methods section. Rats received *DNase I* treatment before CPB (1 × *DNase*, n = 7) or a second *DNase I* dose before reperfusion (2 × *DNase*, n = 8). Animals without *DNase I* treatment served as control (n = 7). Plasma levels of IL-6, TNF-α, IFN-ɣ and IL-10 were quantified using Procartaplex Multiplex Assays before CPB (T1), before (T2) and after (T3) reperfusion. Rats that received two doses of *DNase I* showed significant reduction of IL-6 at the end of reperfusion (T3).*P < 0.05.

**Figure 4:**
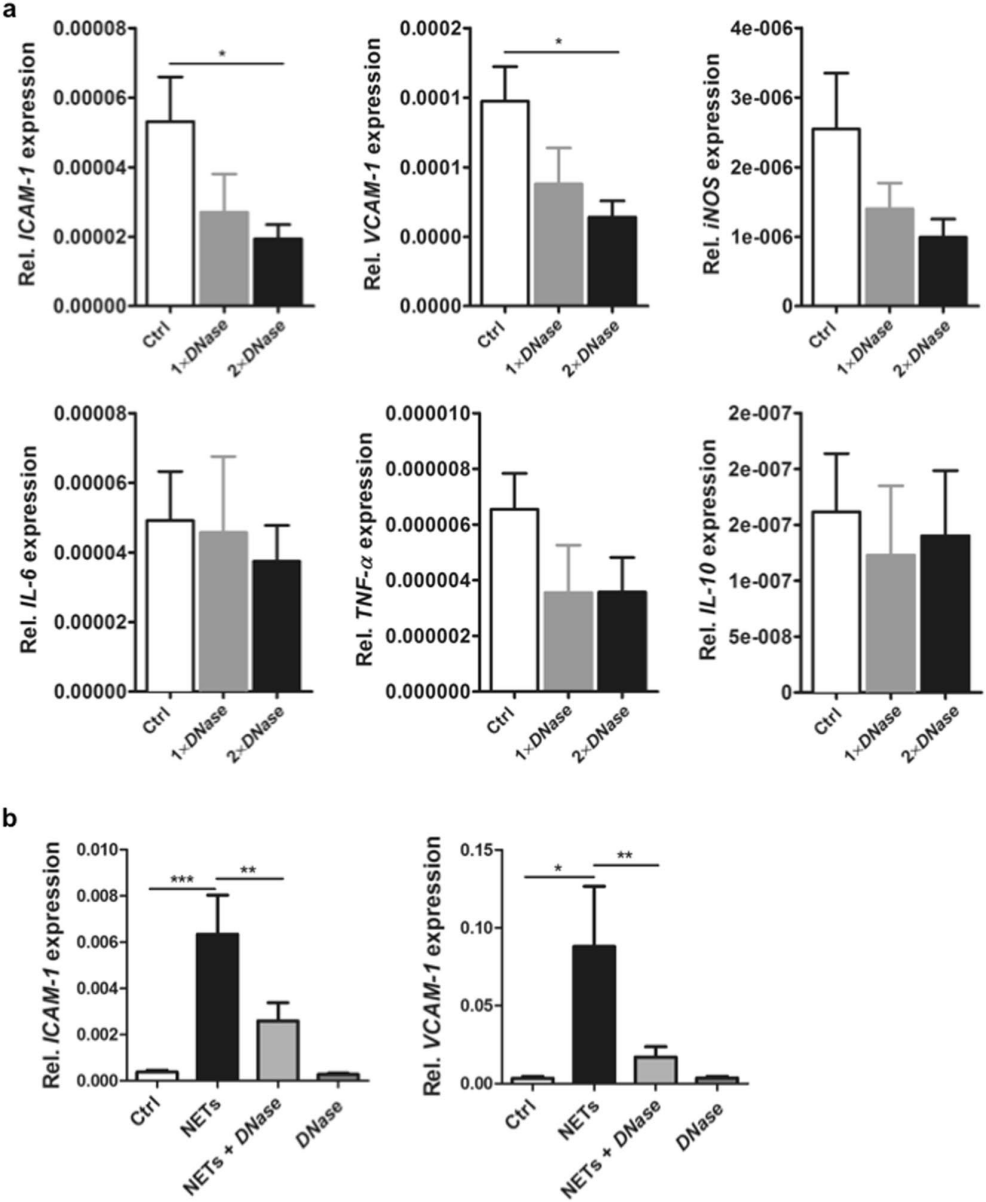
Effects of *DNase I* on endothelial activation. (**a**) Aortas were collected from rats that underwent cardiopulmonary bypass (CPB) with deep hypothermic cardiac arrest (DHCA) at the end of surgery. Relative expression levels of intercellular adhesion molecule-1 (*ICAM-1)*, vascular cell adhesion molecule-1 (*VCAM-1)*, inducible NO synthase (*iNOS)*, *IL-6*, *TNF-α*, and *IL-10* in aortic tissue were analyzed by quantitative real-time PCR. 18 S rRNA was used to normalize the data. Two-time *DNase I* administration (before CPB and before reperfusion) significantly reduced the aortic expression of *ICAM-1* and *VCAM-1*. *P < 0.05. (**b**) HUVECs (3 × 10^5^) were incubated with NETs (1000 ng/ml), *DNase I*-digested NETs or *DNase I* alone for 6 h, followed by RNA isolation and relative quantification of *ICAM-1* and *VCAM-1* expression by qPCR. Significant upregulation of gene expression was observed in the presence of NETs. This effect was abolished after incubation of cells with *DNase I*-degraded NETs (n = 4). *P < 0.05; **P < 0.01; ***P < 0.001.

To evaluate the impact of *DNase I* administration on CPB-induced inflammation, plasma levels of IL-6, TNF-α, IFN-ɣ and IL-10 were quantified (Fig. 3). All cytokines markedly increased at T3 and significant reduction of IL-6 was found in rats that received two doses of *DNase I* (4-fold, *P* = 0.04 vs. Control). No further significant changes were detected. As the endothelium contributes to leukocyte recruitment, the expression of inflammatory markers in the aortic endothelium was examined. *DNase I* (two doses) significantly downregulated vascular expression of *ICAM-1* and *VCAM-1* (2.2-fold and 1.9-fold vs. Control, *P* < 0.05), but did not change the expression of inducible nitric oxide synthase (*iNOS)*, which is upregulated by pro-inflammatory cytokines and implicated in endothelial dysfunction^23^, as well as *IL-6*, *TNF-α* or *IL-10*, respectively (Fig. 4a). To further evidence that cfDNA comprise mainly NETs-derived DNA, which in turn causes endothelial activation, we performed additional *in vitro* experiments. Treatment of human endothelial cells with NETs augmented the expression of *ICAM-1* and *VCAM-1* (Fig. 4b). However, these effects were not observed when cells were treated with *DNase I*-digested NETs or *DNase I* alone, respectively, demonstrating again that NETs degradation by *DNases* indeed counteracts endothelial activation.

**Figure 5:**
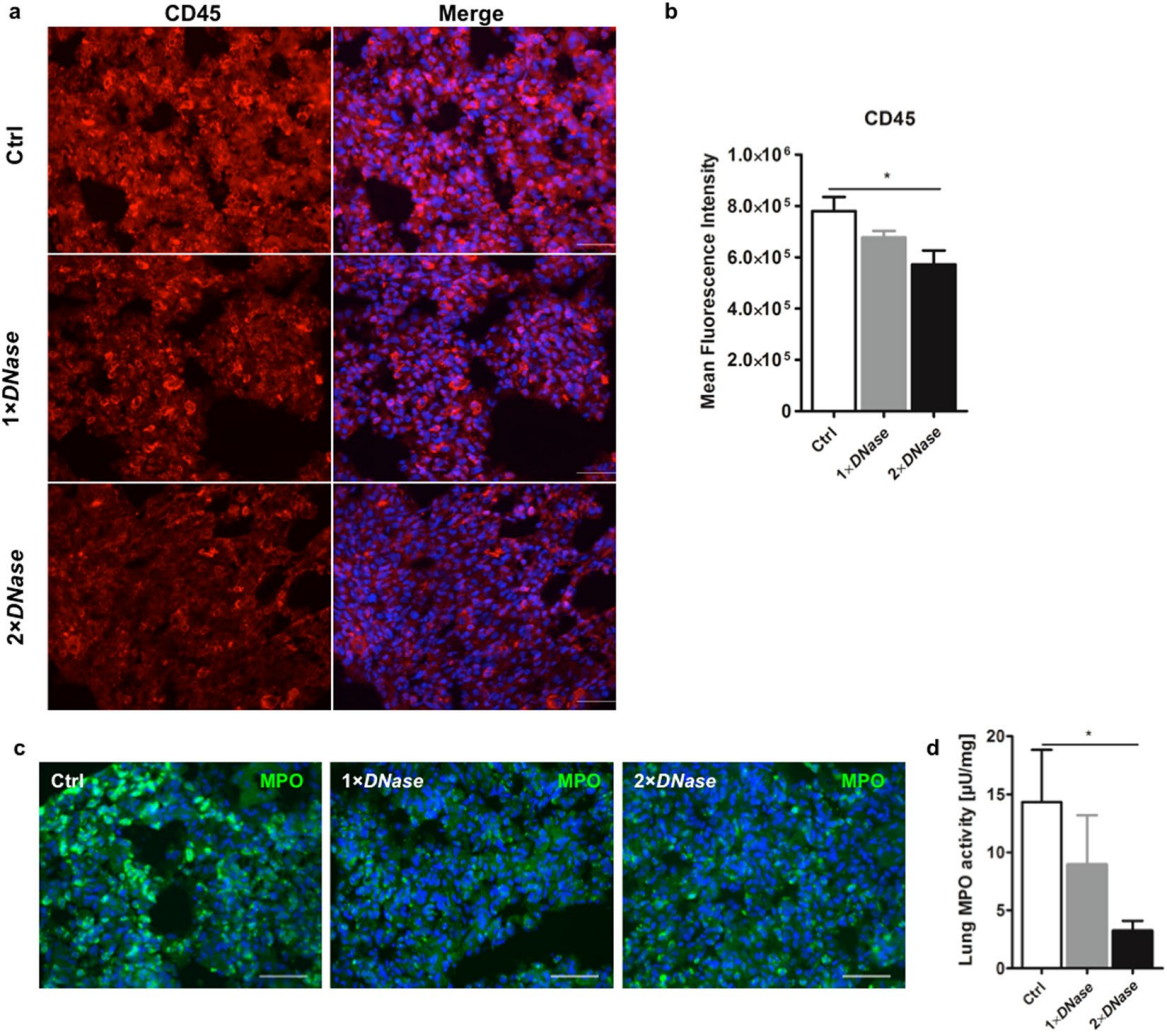
Impact of *DNase I* on leukocyte extravasation. Rats with or without *DNase I* treatment that underwent cardiopulmonary bypass (CPB) with deep hypothermic cardiac arrest (DHCA) were sacrificed and lung tissue was collected at the end of surgery. (**a**) Immunofluorescence detection of CD45 in lung tissue of control rats (Ctrl) and those treated with *DNase I* (1 × *DNase I*, 2 × *DNase I*). Three animals per group were examined. Representative images are depicted. Scale bar: 50 µm. (**b**) Quantification of CD45 fluorescence expressed as mean fluorescence intensity. Ten random fields were examined from each specimen at 400 × magnification. *DNase I* treatment significantly reduced the number of CD45-positive cells.*P < 0.05. (**c**) Immunofluorescence detection of MPO in lung tissue. Three animals per group were examined. Representative images are depicted. Scale bar: 50 µm. (**d**) Neutrophil MPO activity was determined in lung homogenates. Two-time *DNase I* administration before CPB and before reperfusion diminished MPO activity in lungs. *P < 0.05.

Next, we investigated if diminished endothelial activity upon *DNase I* administration is consequently reflected by a reduced number of extravasated leukocytes. In this regard, we found a significant reduction in the number of infiltrated CD45-positive leukocytes in lung tissue of *DNase I*-treated rats compared to controls (Fig. 5a,b) supporting diminished leukocyte recruitment. Furthermore, the number of MPO-positive cells (Fig. 5c) as well as MPO activity (Fig. 5d) in lungs of *DNase I*-treated rats were significantly reduced. Overall, the effects of *DNase I* were more pronounced when two doses were applied.

## Discussion

Despite major improvements in circuit design, oxygenators and the advent of heparin-bonded surfaces, the inflammatory response to CPB remains a clinical concern. There have been several attempts to attenuate postoperative cytokine release including anti-inflammatory therapy^24^, modifications in the bypass circuit^25^ or changing temperature management in CPB^26^.

**Figure 6:**
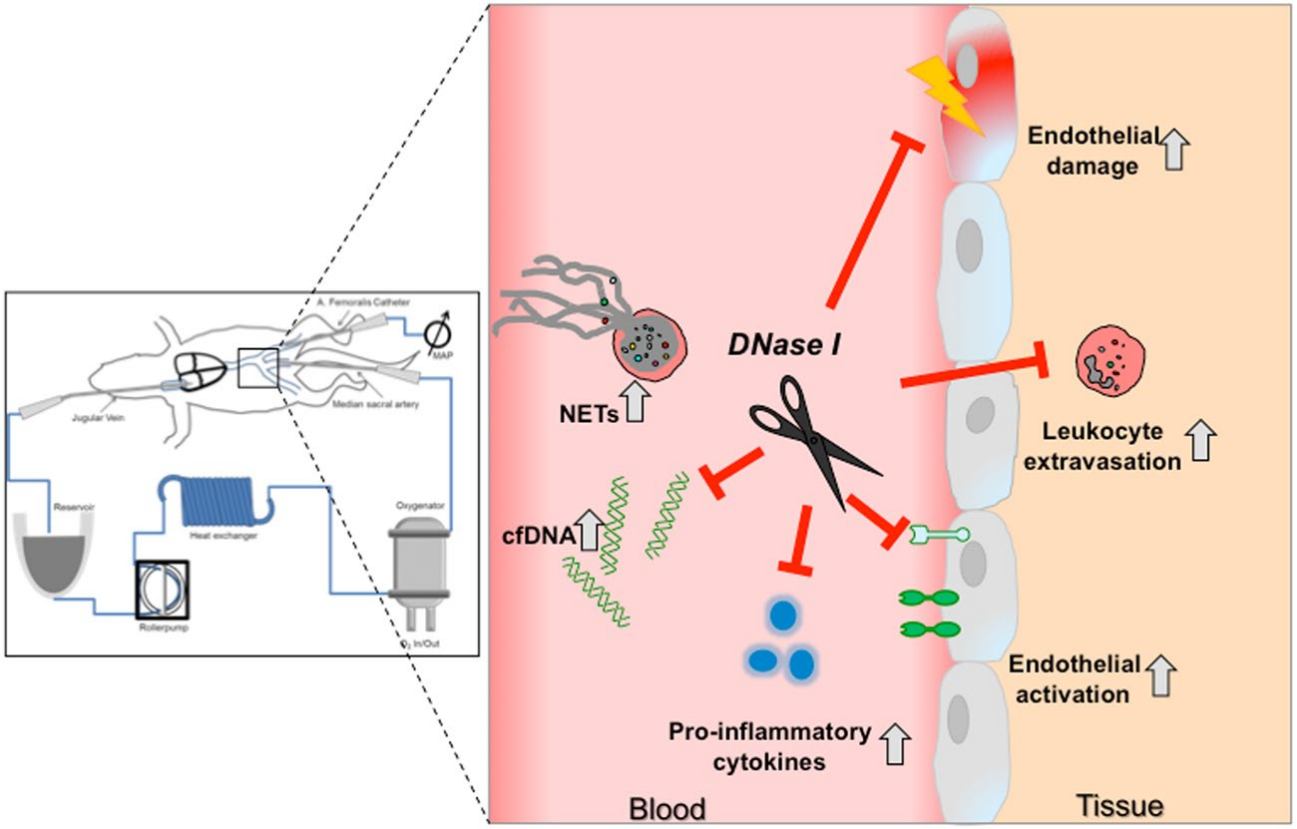
Visual summary of *DNase I*-mediated vasoprotection during CPB. Systemic application of *DNase I* during CPB efficiently degraded cfDNA/NETs released by activated neutrophils and prevented reperfusion-induced cellular damage. *DNase I* improved endothelial function, reduced endothelial activation and leukocyte extravasation.

Activation of neutrophils and the consecutive release of NETs seem to play a pivotal role in inflammatory reactions. NETs were first discovered in 2004 as a unique form of cell death, distinct from necrosis or apoptosis^27^. Although NETs were originally believed to trap, immobilize and kill microbes, recent studies have revealed novel roles of NETs in coagulation activation and tissue damage^4,5^. Additionally, I/R-triggered cfDNA/NETs release has already been reported^18,28^. Here, cfDNA/NETs levels were found to be strongly increased after the reperfusion of rats that underwent CPB with DHCA. Qi *et al*. showed a significant correlation of neutrophil activation (S100A12) and elevation in serum cfDNA, suggesting a major contribution of neutrophil-derived cfDNA to elevated serum cfDNA concentrations^3^. As we did not find any alteration in cardiac troponin levels, increase in plasma cfDNA might largely derive from NETing neutrophils and probably to a small extend from necrotic tissue. Besides reactive oxygen species, NETs-associated components including histones, elastase and MPO may trigger endothelial injury, contributing to tissue injury in major organs^3,16,29,30^. Indeed, high levels of soluble ICAM-1 were detected after reperfusion accompanied by impaired vasorelaxation in control animals indicating vascular injury. In line with these results, in a recent pilot study with patients who underwent cardiac surgery with CPB, we found significantly elevated cfDNA/NETs levels immediately after surgery that sustained until at least day 5 post-surgery^6^. Of note, plasma cfDNA/NETs levels increased with the duration of CPB support and high cfDNA/NETs concentrations strongly correlated with levels of soluble ICAM-1, which is a valuable biomarker of endothelial activation and damage. It might therefore be speculated, that enzymatic cfDNA/NETs degradation might represent a feasible strategy to limit collateral damage after on-pump cardiac surgery. In this regard, Pulmozyme® (recombinant human *DNase I* (*rhDNase I*)), has already been used in multiple animal models of thrombosis^4,31^, as well as in various models of I/R injury^28^. Treatment with *DNase I* significantly reduces inflammation and thrombosis as evidenced by attenuated oxidative stress, apoptosis, and tissue damage after intestinal I/R injury^28,32^. Although *DNase I* efficiently degrades cfDNA it has been suggested that it does not eliminate extracellular histones, which are also known to possess cytotoxic activity^33^. Here, *DNase I* application before CPB prevented cfDNA/NETs increase after DHCA but failed to inhibit cfDNA/NETs elevation after reperfusion, which might be explained by the relatively short half-life of *DNases* due to their rapid inactivation by serum components, such as monomeric actin^22^. In contrast, a second dose of *DNase I* before reperfusion markedly abolished a later increase in cfDNA/NETs. As plasma cfDNA levels were found to largely reach baseline levels, our results strongly support the feasibility of *i.v*. administrated *DNase I* for cfDNA/NETs degradation. More importantly, cfDNA/NETs degradation, especially before reperfusion, significantly improved vessel function arguing against toxic side effects of histones or NETs-associated proteases, respectively. *DNase I* significantly diminished plasma IL-6 levels and leukocyte infiltration in the lung, as evidenced by a reduced number of CD45-positive cells and diminished pulmonary MPO activity, being in line with the findings of Merza *et al*., who showed that NETs degradation reduces inflammation and neutrophil recruitment^34^. The protective effects of *DNase I* after *i.v*. administration are summarized in Fig. 6. We propose that CPB and reperfusion trigger neutrophil activation and consequently the release of NETs. In accordance with data reported by Folco *et al*., we demonstrate that NETs promote endothelial activation *in vivo* and *in vitro* by upregulating the expression of adhesion molecules and pro-inflammatory cytokines^15^. However, in contrast to the results reported by Saffarzadeh *et al*., we did not find evidence for NETs-mediated cytotoxic effects on human endothelial cells (unpublished results)^16^. This finding might be explained by the concentration of NETs used in our experiments, which was 3- to 10-fold lower than the concentration found to have a toxic effect. Although *in vivo* effects of cfDNA/NETs could not be fully reproduced *in vitro*, the improvement of endothelial function in *DNase I*-treated rats strongly supports the hypothesis that NETs damage the endothelium. Here, *DNase I* efficiently degraded CPB-induced cfDNA/NETs, thus reducing endothelial cell activation and tissue/endothelial injury.

However, our study has several limitations that need to be considered. First, cardiac arrest was induced by deep hypothermia and not by cardioplegia. Our well-established rat model incorporates DHCA, which is still successfully used in modern aortic arch surgery. The exposition of organs to hypothermia reduces energy consumption and arrests cellular metabolism thus ensuring tissue integrity, but coincidentally it induces global I/R. Our standardized model can reproduce pathophysiological alterations that are associated with SIRS and I/R and helps to investigate novel therapeutic strategies in a preclinical setting^35^. Secondly, due to restrictions of our experimental proposal, animals had to be sacrificed at the end of CPB. Therefore, it would be worthwhile to investigate long-term effects of *DNase I* in this animal model. Nevertheless, the results of this study provide strong evidence for anti-inflammatory, vasculoprotective effects of *i.v*. administrated *DNase I* although efficacy in surgical patients was not evidenced and should be further evaluated. Altogether, this strategy might represent a possible therapeutic tool for the treatment of high-risk cardiac patients with on-pump surgery.

## Methods

### Animals and study design

This study was approved by the local authority LANUV (Landesamt für Natur, Umwelt und Verbraucherschutz NRW; AZ84-02.04.2014.A103) and carried out in accordance with German and European guidelines of laboratory animal care. 24 male Wistar rats weighing between 510 and 525 g (Janvier Breeding Center, France) were randomly assigned to three experimental groups: one control group undergoing CPB with DHCA (group 1), one group undergoing CPB with DHCA that received an *i.v*. bolus of *DNase I* (Pulmozyme®, Roche, 5 mg/kg body weight) before CPB (group 2), and one group undergoing CPB with DHCA that received *DNase I* before CPB and a second bolus after rewarming/before reperfusion, respectively (group 3). This *DNase I* dose was previously found to effectively degrade plasma cfDNA/NETs^18,36^. Two animals with low haemoglobin concentration during the procedure (Hb < 5.0 mg/dL) caused by a diffuse bleeding at the insertion site of the arterial cannula developed a catecholamine-refractory hypotension and were subsequently excluded from the study. The study was conducted with 7 animals in groups 1 and 2 respectively, and 8 animals in group 3.

### Surgical procedure

Surgical procedures were performed as previously described^35^. In brief, animals were intubated and underwent inhalative anaesthesia with 2.5% isoflurane. After animal cannulation and before CPB start, *DNase I* was administrated *i.v*. Control animals (group 1) received vehicle solution (0.9% NaCl). Rats were connected to the CPB device (M. Humbs, Valley, Germany) consisting of an oxygenation device, a heat exchanger and a roller pump for flow support. Blood flow was directed from the jugular vein through silicon tubes to the CPB device and back to the corporal circulation via the tail artery. After systemic cooling of the animal, DHCA was induced at 16 °C body core temperature for 45 min. Then, CPB was restarted and the rats were subjected to 40 min of rewarming. Before reperfusion, animals included in group 3 received a second bolus of *DNase I*, whereas animals included in groups 1 and 2 received the equivalent volume of 0.9% NaCl. The rats were reperfused for another 60 min before CPB was terminated and animals were euthanized. One part of the thoracic aorta was used for vascular function studies, the other parts were stored at −80 °C for further analyses. Arterial blood samples were collected before CPB (T1), before reperfusion (T2) and at the end of reperfusion (T3). Samples were centrifuged at 1,000 × *g* for 20 min at 4 °C and plasma supernatants were stored at −80 °C until further use.

Baseline physiological parameters, MAP, rectal temperature, electrocardiogram and peripheral oxygen saturation were continuously monitored during the CPB procedure. If necessary, sodium bicarbonate (NaBiC 8.4%, B. Braun), trometamol (TRIS 36.34%, B. Braun) or CO_2_ were applied or an altered ventilation scheme was used. In the rewarming and reperfusion phases at body temperatures of at least 30 °C norepinephrine (Arterenol, Sanofi-Aventis) was applied in case of MAP values lower than 40 mm HG.

### Measurement of endothelial function

The thoracic aorta was cut into four segments (4 mm width). Ring segments were mounted between two stainless steel hooks in individual organ baths (Hugo Sachs Elektronik, Germany) containing 10 ml of modified Krebs-Henseleit buffer (in mM: 118 NaCl, 5 KCl, 1.2 KH_2_PO_4_, 1.2 MgSO_4_, 24.9 NaHCO_3_, 2.5 CaCl_2_, and 11.0 glucose; pH 7.4) at 37 °C bubbled with 95% O_2_/5% CO_2_. The rings were equilibrated for 40 min under a resting tension of 1 g to allow development of a stable basal tone. Stimulation of rings with 60 mM KCl was repeated three times until contractile responses were stable and uniform. Indomethacin (10 µM) was present in the experiments to inhibit prostaglandin synthesis. To inhibit the activity of endothelial nitric oxide synthase (eNOS), some rings were pretreated with 100 µM N^G^-nitro-L-Arginine (L-NNA). All vessels were preconstricted with 0.1 µM phenylephrine (PE). Ligand-stimulated receptor-mediated NO bioavailability was assessed by a concentration-dependent relaxation to acetylcholine (ACh, 1 nM-10 µM). To study endothelium-independent vasorelaxation, aortic rings preconstricted with PE were treated with the NO donor S-Nitroso-N-acetyl-D,L-penicillamine (SNAP; 0.1 nM-10 µM).

Relaxation responses to ACh and SNAP were expressed as percentage of relaxation from a submaximal PE-induced constriction and concentration–response curves were obtained.

### Analysis of electrolytes, gases and cell counts in arterial blood

The analysis of electrolytes, gases and cell counts in arterial blood samples was performed using the ABL 700 (Radiometer Copenhagen) and Vet abc (Scil animal care) devices.

### Preparation of NETs

To induce NETs, human neutrophils were plated in 6-well plates and stimulated with 100 nM phorbol myrostate acetate (PMA) for 3.5 h. Supernatants were carefully discarded and NETs were partially digested by *Alu I* (4 U/ml, New England Biolabs) for 20 min at 37 °C, followed by centrifugation at 300 × g for 5 min. Supernatants containing NETs were collected and stored at −80 °C until use. The formation of NETs was confirmed by measuring peroxidase activity in culture supernatants as previously described^37^. NETs concentration was quantified using a NanoDrop spectrophotometer (Thermo Fisher).

### Cell culture experiments

Human umbilical endothelial cells (HUVECs) were kindly provided by Dr. Paola Zigrino (Department of Dermatology and Venereology, University Hospital Cologne). Cells (3 × 10^5^) were cultured in 6-well plates in EGM-2 medium supplemented with EGM-2 SingleQuots (Lonza) and treated with NETs for 6 h and 24 h, respectively. In parallel experiments, cells were incubated in the presence of NETs pre-digested with recombinant *DNase I* (100 U/ml, Roche) or *DNase I* alone.

### Quantification of plasma cfDNA levels

Plasma levels of cfDNA were quantified by Quant-iT Pico Green dsDNA assay (Invitrogen) as recently reported^6^.

### Quantification of systemically circulating cytokines, ICAM-1 and injury markers

A rat Multiplex Immunoassay (ProcartaPlex 4 Plex, Thermo Fisher) was used to quantify cytokines in plasma samples and a Simplex Kit (Rat ICAM-1 Procarta Plex Simplex, Thermo Fisher) for ICAM-1 quantification. All samples were analyzed using Luminex 200 System (Thermo Fisher).

Plasma concentrations of all other organ-specific injury markers were determined at the Central Institute of Clinical Chemistry and Laboratory Diagnostics of Düsseldorf University Hospital using automated analysers from Roche Diagnostics.

### Measurement of *DNase I* plasma activity

*DNase I* activity in plasma samples was measured by the method already reported^6^.

### Determination of tissue and plasma MPO activity

Immediately after euthanization, the lungs were removed and snap frozen in liquid nitrogen. MPO activity in lung tissues was determined by using the MPO Activity Assay Kit II (PromoCell) according to the manufacturer’s instructions. Fluorescence-based kinetic measurements at excitation and emission wavelengths of 485 nm and 530 nm were performed using a microplate reader (Victor X3, Perkin Elmer). Units of MPO activity per 1 min were calculated from a standard curve using fluorescein standard.

### Immunofluorescence staining

Frozen lung tissue samples of rats were sectioned at 7 µm intervals and fixed with 4% paraformaldehyde. For the detection of MPO-positive cells (neutrophils, monocytes), sections were permeabilized with 0.2% Triton X-100 and incubated with 5% normal goat serum + 0.5% BSA + 0.2% TritonX-100 in PBS in order to block unspecific binding. Sections were further incubated with anti-MPO antibody (1:100, Abcam) for 1 h followed by additional incubation with Alexa Fluor 488-conjugated goat anti-rabbit IgG (1:1000, Cell Signaling Technology) overnight at 4 °C.

For the detection of leukocytes, a mouse monoclonal anti-rat CD45 antibody was used (clone OX-1, 1:20, BD Pharmingen). Then, slides were further incubated with Alexa Fluor 555-conjugated goat anti-mouse IgG (H + L) secondary antibody (1:1000, Thermo Fisher) for 1 h at room temperature. Nuclei were counterstained with 4′,6-diamidino-2-phenyl indole (DAPI) and slides were mounted in fluorescence mounting medium (Dako). Tissue sections were examined using an inverted microscope (Eclipse Ti-U 100, Nikon). Signal intensity of CD45 immunolabeling in ten random fields was quantified as mean value and averaged using the Image J analysis software.

### Quantitative real-time PCR (qPCR)

RNA was extracted from thoracic aorta tissue using TRIzol (Invitrogen) and the RNeasy Mini Kit (Qiagen). Reverse transcription to cDNA was performed using Quantitect Reverse Transcription Kit (Qiagen). For qPCR, the GoTaq PCR Master Mix (Promega) and self-designed primers against mRNA transcripts encoding rat ICAM-1, VCAM-1, TNF-α, IL-6, IL-10, iNOS as well as human ICAM-1 and VCAM-1 have been used. Expression levels for each mRNA were normalized to the mRNA level of 18 S rRNA (rat) or GAPDH (human), respectively. The relative mRNA expression level was calculated using the comparative threshold cycle method (∆C_T_).

### Western blot analyses

Proteins were extracted from thoracic aorta tissue using Cell Lysis Buffer (Cell Signaling) supplemented with proteinase and phosphatase inhibitors (Complete Mini and PhosSTOP, Roche). For Western blot, 50 µg of total protein was loaded on 7% polyacrylamide gels and blotted onto nitrocellulose membranes via tank blot. Expression levels of total eNOS and eNOS phosphorylated at Ser1177 were analysed using antibodies from BD Biosciences (#610297) and Cell Signaling Technology (#9570), respectively. Protein bands were visualized using a chemiluminescence system (Thermo Fisher). Band intensities were quantified using ImageJ software (National Institutes of Health).

### Statistical analyses

Statistical analyses were performed using GraphPad Prism 5 software (GraphPad Software Inc., San Diego, CA, USA). Data are presented as Data are presented as mean ± SEM. Data sets were assessed for normality using the Kolmogorov-Smirnov test. Normally distributed unpaired data of multiple groups were analyzed using one-way ANOVA with Newman Keuls post-hoc test. The Kruskal–Wallis test was used for nonparametric values. Correlations were evaluated by the Pearson correlation coefficient (*r*). *P*-values < 0.05 were considered as statistically significant.

## Supplementary information

Supplementary information

## Data Availability

All data generated or analyzed in this study are available from the corresponding author on reasonable request. All experiments were performed in accordance with relevant guidelines and regulations.

## References

[1] Dabbous A, Kassas C, Baraka A (2003). The inflammatory response after cardiac surgery. Middle East J Anaesthesiol.

[2] Caputo Massimo, Mokhtari Amir, Miceli Antonio, Ghorbel Mohamed T., Angelini Gianni D., Parry Andrew J., Suleiman Saadeh M. (2014). Controlled reoxygenation during cardiopulmonary bypass decreases markers of organ damage, inflammation, and oxidative stress in single-ventricle patients undergoing pediatric heart surgery. The Journal of Thoracic and Cardiovascular Surgery.

[3] Qi Y (2016). Perioperative Elevation in Cell-Free DNA Levels in Patients Undergoing Cardiac Surgery: Possible Contribution of Neutrophil Extracellular Traps to Perioperative Renal Dysfunction. Anesthesiol Res Pract.

[4] Fuchs TA (2010). Extracellular DNA traps promote thrombosis. Proc Natl Acad Sci USA.

[5] Mangold A (2015). Coronary neutrophil extracellular trap burden and deoxyribonuclease activity in ST-elevation acute coronary syndrome are predictors of ST-segment resolution and infarct size. Circ Res.

[6] Paunel-Gorgulu A (2017). cfDNA correlates with endothelial damage after cardiac surgery with prolonged cardiopulmonary bypass and amplifies NETosis in an intracellular TLR9-independent manner. Sci Rep.

[7] Urban CF (2009). Neutrophil extracellular traps contain calprotectin, a cytosolic protein complex involved in host defense against Candida albicans. PLoS Pathog.

[8] Clark SR (2007). Platelet TLR4 activates neutrophil extracellular traps to ensnare bacteria in septic blood. Nat Med.

[9] Gupta AK (2010). Activated endothelial cells induce neutrophil extracellular traps and are susceptible to NETosis-mediated cell death. FEBS Lett.

[10] Neeli I, Khan SN, Radic M (2008). Histone deimination as a response to inflammatory stimuli in neutrophils. J Immunol.

[11] Czaikoski PG (2016). Neutrophil Extracellular Traps Induce Organ Damage during Experimental and Clinical Sepsis. PLoS One.

[12] Peer V, Abu Hamad R, Berman S, Efrati S (2016). Renoprotective Effects of DNAse-I Treatment in a Rat Model of Ischemia/Reperfusion-Induced Acute Kidney Injury. Am J Nephrol.

[13] Duffy MJ (2011). Impaired endothelium-dependent vasodilatation is a novel predictor of mortality in intensive care. Crit Care Med.

[14] Kunes P (2007). The inflammatory response in cardiac surgery. An up-to-date overview with the emphasis on the role of heat shock proteins (HSPs) 60 and 70. Acta Medica (Hradec Kralove).

[15] Folco EJ (2018). Neutrophil Extracellular Traps Induce Endothelial Cell Activation and Tissue Factor Production Through Interleukin-1alpha and Cathepsin G. Arterioscler Thromb Vasc Biol.

[16] Saffarzadeh M (2012). Neutrophil extracellular traps directly induce epithelial and endothelial cell death: a predominant role of histones. PLoS One.

[17] Silk E, Zhao H, Weng H, Ma D (2017). The role of extracellular histone in organ injury. Cell Death Dis.

[18] Wang S (2018). DNase-1 Treatment Exerts Protective Effects in a Rat Model of Intestinal Ischemia-Reperfusion Injury. Sci Rep.

[19] Savchenko AS (2014). VWF-mediated leukocyte recruitment with chromatin decondensation by PAD4 increases myocardial ischemia/reperfusion injury in mice. Blood.

[20] Vogel B, Shinagawa H, Hofmann U, Ertl G, Frantz S (2015). Acute DNase1 treatment improves left ventricular remodeling after myocardial infarction by disruption of free chromatin. Basic Res Cardiol.

[21] Engels M (2014). A cardiopulmonary bypass with deep hypothermic circulatory arrest rat model for the investigation of the systemic inflammation response and induced organ damage. J Inflamm (Lond).

[22] Prince WS (1998). Pharmacodynamics of recombinant human DNase I in serum. Clin Exp Immunol.

[23] Cortese-Krott MM (2014). Zinc regulates iNOS-derived nitric oxide formation in endothelial cells. Redox Biol.

[24] Greilich PE (2003). Antifibrinolytic therapy during cardiopulmonary bypass reduces proinflammatory cytokine levels: a randomized, double-blind, placebo-controlled study of epsilon-aminocaproic acid and aprotinin. J Thorac Cardiovasc Surg.

[25] Defraigne JO, Pincemail J, Larbuisson R, Blaffart F, Limet R (2000). Cytokine release and neutrophil activation are not prevented by heparin-coated circuits and aprotinin administration. Ann Thorac Surg.

[26] Schmitt KR (2016). Hypothermia During Cardiopulmonary Bypass Increases Need for Inotropic Support but Does Not Impact Inflammation in Children Undergoing Surgical Ventricular Septal Defect Closure. Artif Organs.

[27] Brinkmann V (2004). Neutrophil extracellular traps kill bacteria. Science.

[28] Boettcher M (2017). Therapeutic targeting of extracellular DNA improves the outcome of intestinal ischemic reperfusion injury in neonatal rats. Sci Rep.

[29] Liu S (2016). Neutrophil extracellular traps are indirectly triggered by lipopolysaccharide and contribute to acute lung injury. Sci Rep.

[30] Zakkar M, Guida G, Suleiman MS, Angelini GD (2015). Cardiopulmonary bypass and oxidative stress. Oxid Med Cell Longev.

[31] Brill A (2012). Neutrophil extracellular traps promote deep vein thrombosis in mice. J Thromb Haemost.

[32] Boettcher M (2017). Degradation of Extracellular DNA by DNase1 Significantly Reduces Testicular Damage After Testicular Torsion in Rats. Urology.

[33] Allam R, Darisipudi MN, Tschopp J, Anders HJ (2013). Histones trigger sterile inflammation by activating the NLRP3 inflammasome. Eur J Immunol.

[34] Merza M (2015). Neutrophil Extracellular Traps Induce Trypsin Activation, Inflammation, and Tissue Damage in Mice With Severe Acute Pancreatitis. Gastroenterology.

[35] Pinto A (2016). Modulation of Immunologic Response by Preventive Everolimus Application in a Rat CPB Model. Inflammation.

[36] Meng W (2012). Depletion of neutrophil extracellular traps *in vivo* results in hypersusceptibility to polymicrobial sepsis in mice. Crit Care.

[37] Eghbalzadeh K (2019). Compromised Anti-inflammatory Action of Neutrophil Extracellular Traps in PAD4-Deficient Mice Contributes to Aggravated Acute Inflammation After Myocardial Infarction. Front Immunol.

