# Supplementary material for: Targeting of cell-free DNA by DNase I diminishes endothelial dysfunction and inflammation in a rat model of cardiopulmonary bypass
Source: Sci Rep. 2019 Dec 17;9:19249. doi: 10.1038/s41598-019-55863-8 (PMC6917735; doi:10.1038/s41598-019-55863-8)

**Targeting of cell-free DNA by *DNase I* diminishes endothelial dysfunction**

**and inflammation in a rat model of cardiopulmonary bypass**

Carolyn Weber^1#^*, Alexander Jenke^2#^, Vasilena Chobanova^1^, Mariam Yazdanyar^2^, Agunda Chekhoeva^2^, Kaveh Eghbalzadeh^1^, Artur Lichtenberg^2^,Thorsten Wahlers^1^, Payam Akhyari^2#^, Adnana Paunel-Görgülü^1#^

^1^Department of Cardiothoracic Surgery, Heart Center of the University of Cologne, Cologne, Germany

^2^Department of Cardiovascular Surgery, Heinrich Heine University Düsseldorf, Düsseldorf, Germany

^#^Both first and senior authors contributed equally to this work

Corresponding author:

*Dr. Carolyn Weber

Department of Cardiothoracic Surgery, University of Cologne,

Kerpener Strasse 62, D-50937 Cologne, Germany

Phone: +49 221 47832441, email: carolyn.weber@uk-koeln.de

**Supplementary Table 1: *DNase I*** does not affect blood cell count, haemoglobin concentration and haematocrit during CPB with DHCA

|  |  | Control | | |  | 1x *DNase* | | |  | 2x *DNase* | | |
| --- | --- | --- | --- | --- | --- | --- | --- | --- | --- | --- | --- | --- |
|  |  | T1 | T2 | T3 |  | T1 | T2 | T3 |  | T1 | T2 | T3 |
| WBC (10^3^/mm^3^) |  | 6.4 ± 2.3 | 4.8 ± 1.5 | 6.3 ± 2.1 |  | 8.8 ± 3.1 | 5.9 ± 1.3 | 6.2 ± 1.7 |  | 6.1 ± 2.2 | 5.6 ± 1.2 | 5.9 ± 1.7 |
| RBC (10^6^/mm^3^) |  | 6.1 ± 1.8 | 4.8 ± 0.3 | 4.7 ± 0.6 |  | 5.7 ± 1.0 | 4.7 ± 0.3 | 4.4 ± 0.7 |  | 5.3 ± 1.0 | 4.7 ± 0.5 | 4.6 ± 0.6 |
| PLT (10^3^/mm^3^) |  | 303.4 ± 183.9 | 332.6 ± 27.9 | 380.4 ± 69.6 |  | 289.0 ± 144.8 | 337.4 ± 41.6 | 395.0 ± 72.0 |  | 344.9 ± 49.8 | 296.0 ± 60.0 | 386.3 ± 53.9 |
| Hb (g/dL) |  | 12.9 ± 1.1 | 9.0 ± 0.6^***^ | 8.3 ± 0.9^***^ |  | 12.7 ± 1.2 | 8.9 ± 0.4^***^ | 8.1 ± 1.1^***^ |  | 12.8 ± 0.9 | 9.1 ± 1.0^***^ | 8.4 ± 1.3^***^ |
| HCT (%) |  | 32.7 ± 9.2 | 25.2 ± 2.5 | 23.9 ± 3.3 |  | 31.8 ± 5,3 | 24.1 ± 1.2 | 22.8 ± 3.8 |  | 28.1 ± 5.7 | 25.1 ± 2.5 | 24.5 ± 3.1 |
| *WBC*, white blood cells; *RBC*, red blood cells; *PLT*, platelets; *Hb*, haemoglobin; *HCT*, haematocrit; *T1*, before CPB; *T2*, after CPB and before reperfusion; *T3*, after reperfusion. *p<0.05; **p<0.01; ***p<0.001 versus T1.  Data are presented as Mean ± SD. No significant intergroup differences were detected. | | | | | | | | | | | | |

**Supplementary Table 2: *DNase I* does not affect blood levels of injury markers during CPB with DHCA**

|  |  | Control | 1x *DNase* | 2x *DNase* |
| --- | --- | --- | --- | --- |
| Creatinin (mg/dL) |  | 0.50 ± 0.17 | 0.45 ± 0.07 | 0.46 ± 0.06 |
| Urea (mg/dL) |  | 47.5 ± 2.8 | 47.5 ± 3.5 | 46.1 ± 4.3 |
| Uric acid (mg/dL) |  | 0.70 ± 0.36 | 0.68 ± 0.51 | 0.56 ± 0.20 |
| Aspartate transaminase (U/L) |  | 225.0 ± 67.8 | 152.6 ± 42.5 | 258.6 ± 183.3 |
| Alanine transaminase (U/L) |  | 76.7 ± 17.3 | 51.4 ± 12.0 | 84.5 ± 68.9 |
| Alpha-amylase (U/L) |  | 1292.9 ± 192.2 | 1306.4 ± 195.9 | 1361.1 ± 181.9 |
| Lactate dehydrogenase (U/L) |  | 1289.2 ± 316.2 | 937.1 ± 280.3 | 1190.1 ± 429.2 |
| Neuron-specific enolase (µg/L) |  | 1.60 ± 0.52 | 1.28 ± 0.39 | 1.27 ± 0.36 |
| Data are presented as Mean ± SD. No significant intergroup differences were detected. | | | | |

**Supplementary Figure Legends**

**Supplementary Fig. S1: *DNase I* does not affect the cardiocirculatory recovery after DHCA. (a)** Heart rate and **(b)** mean arterial pressure (MAP) of control rats (n = 7), rats receiving *DNase I* before CPB (1×*DNase*, n = 7) and those receiving a second *DNase I* dose before reperfusion (2×*DNase*, n = 8) were monitored and recorded throughout the CPB procedure at the indicated times.

**Supplementary Fig. S2: *DNase I* does not affect the compensatory application of norepinephrine after DHCA.** Norepinephrine *(Arterenol)* was applied in the rewarming and reperfusion phase to control rats (n = 7), rats receiving *DNase I* before CPB (1×*DNase*, n = 7) and those receiving a second *DNase I* dose before reperfusion (2×*DNase*, n = 8) in order to prevent a sustained fall of MAP values below 40 mm HG. The administered drug volumes were recorded for each animal.

**Supplementary Fig. S3.**

Cropped images were used in Fig. 2.


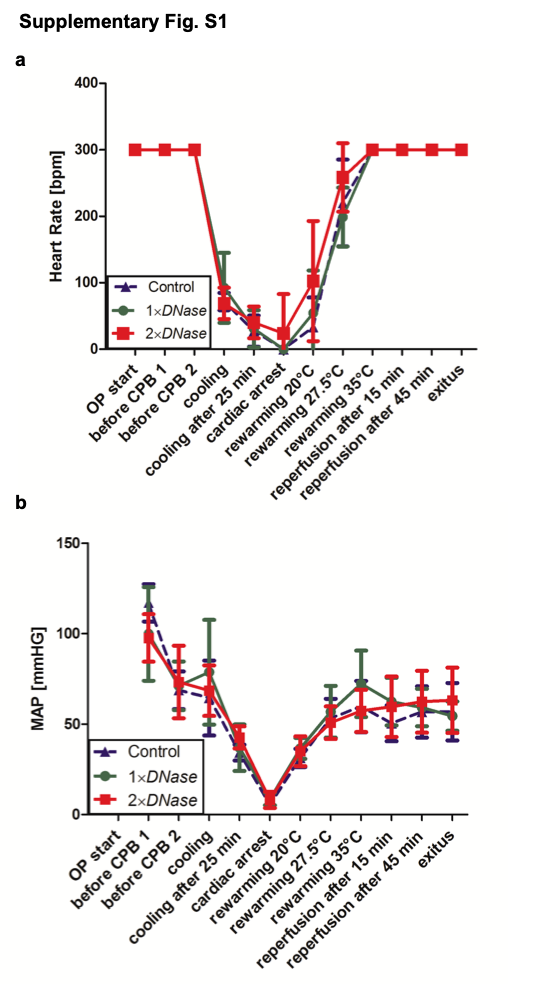


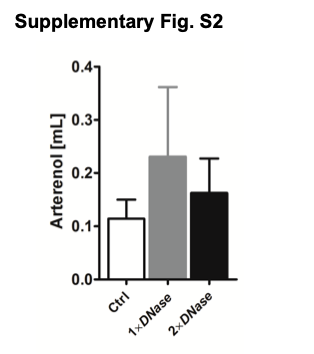


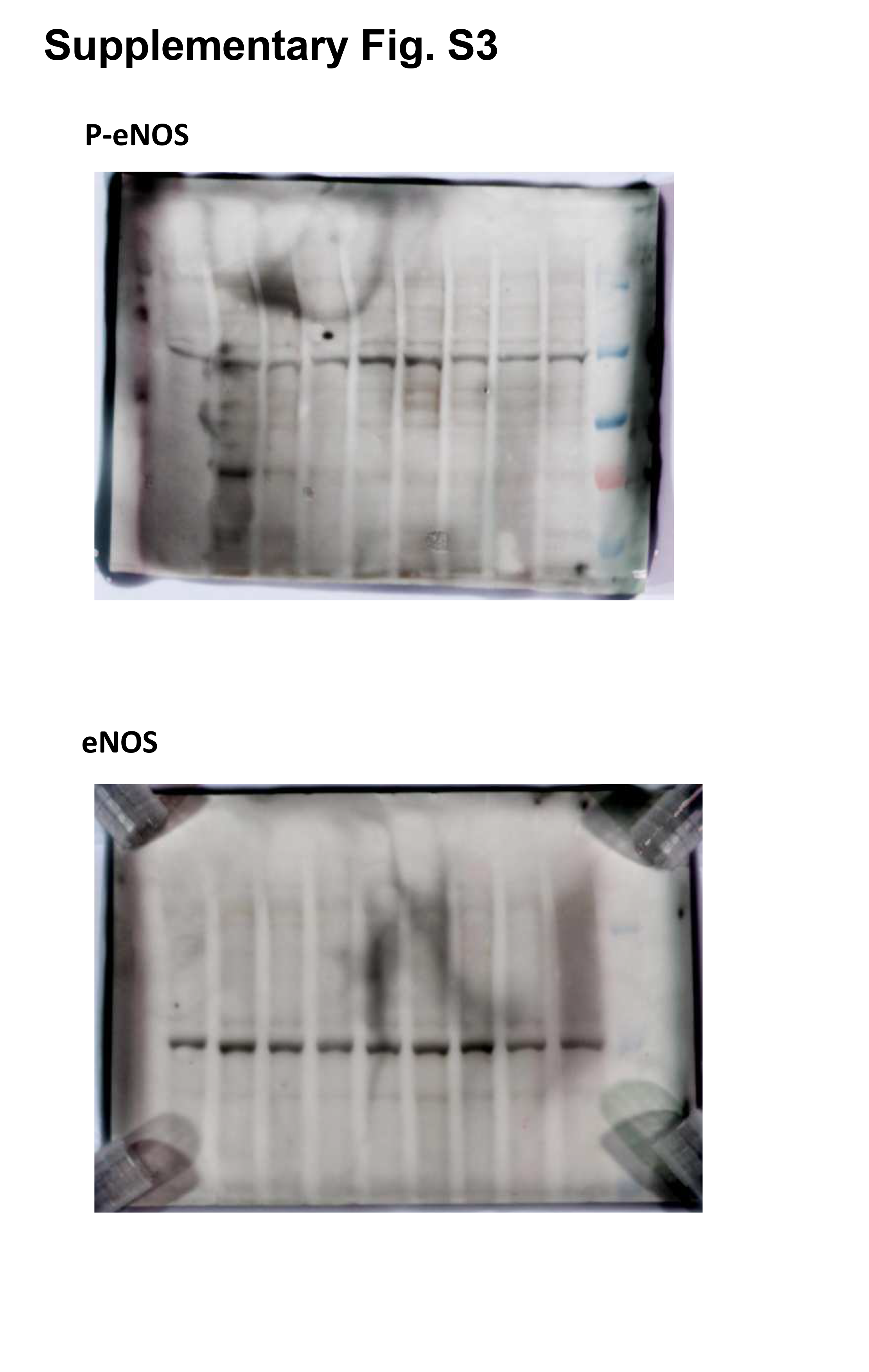

Supplement: Supplementary file 1 — Supplementary information [file 41598_2019_55863_MOESM1_ESM.doc]
